# Impact of nurse-led postoperative education on health outcomes following endovascular aortic repair: a randomized trial

**DOI:** 10.1038/s41598-026-54460-w

**Published:** 2026-05-23

**Authors:** Johan Nilsson, Joakim Nordanstig, Mona Ringdal, Per Skoog, Monica E. Pettersson

**Affiliations:** 1 Institute of Health and Care Sciences, Sahlgrenska Academy, University of Gothenburg, Box 457, 40530 Gothenburg, Sweden; 2 Institute of Medicine, Department of Molecular and Clinical Medicine, University of Gothenburg, Gothenburg, Sweden; 3 Department of Hybrid and Interventional Surgery, Vascular Surgery Unit, Sahlgrenska University Hospital, Gothenburg, Sweden

**Keywords:** Aortic aneurysm, Nursing, Endovascular treatment, Quality of life, Patient education, Nurse-led clinic, Recovery, Cardiology, Diseases, Health care, Medical research

## Abstract

The purpose of this randomized controlled clinical trial was to investigate the health effects of an education-based intervention performed at a nurse-led clinic, for patients who underwent endovascular treatment for abdominal aortic aneurysm. Fifty-four adult patients were randomized to the intervention group (n = 26) or the control group (n = 28). The control group received standard care, and the intervention group received standard care alongside two consultations at a specialist surgical nurse-led clinic. The consultations focused on individual motivational support and education about abdominal aortic aneurysm disease. Data was collected prior to surgery (baseline) and at one, six, and 12 months postoperatively for each group. Outcomes of interest included participants’ responses to the Health Education Impact Questionnaire, responses to the EuroQol Five Dimensions Questionnaire, and responses to four complementary items specific to the scope of this study. Both groups reported overall high ratings of perceived health throughout the study period. While there were no statistically significant differences between the two groups, the control group showed a substantial improvement in perceived understanding of their diagnosis over time. This randomized controlled trial did not find any significant health benefits as compared to standard treatment.

*Trial registration* ClinicalTrials.gov (19/05/2025 NCT06986252). Retrospectively registered.

## Background

Abdominal aortic aneurysm (AAA) is a predominantly symptom-free, but potentially fatal disease. The prevalence of AAA is between 4 and 8% of the population internationally, with a prevalence of 1–7% in Sweden^1^. Between 21 and 33 per 100,000 people suffer from ruptured AAA each year in Sweden and the majority of this population are males in their mid-seventies^2^.

Currently, the only method to prevent AAA rupture is to identify the AAA before rupture occurs, which can be done by several vascular imaging modalities. Treatment of AAA is primarily determined by the diameter and location of the aneurysm, and clinical management can be either conservative observation or surgical intervention. The main indication for surgical intervention is an aortic diameter of 55 mm or greater for males or an aortic diameter of 50 mm or greater for females^3^. Advancements in endovascular surgical techniques and the development of newer AAA implants has led to a shift from open surgery to endovascular repair (EVAR)^3^. EVAR has a lower risk of perioperative complications and a shorter postoperative recovery time than open surgery, and EVAR patients also report an initially higher postoperative quality of life during the first postoperative months^4,5^. Standard care for EVAR includes postoperative follow-up at one month and one year after surgery, as well as annual CT or ultrasound imaging to identify any postoperative complications, such as stent graft migration or endoleak, that may require invasive reinterventions^6^.

Risk factors for AAA, like those for atherosclerosis, are often lifestyle related; the most impactful risk factors are smoking, hypertension, and hyperlipidemia^3^. While these risk factors can be treated with preoperative interventions, they often require prolonged, and in some cases life-long, interventions to mitigate the risk for long-term comorbidity and mortality^7^. Educational interventions have been demonstrated to improve health-related quality of life and adherence to self-care behaviours in the context of diabetes management and cardiovascular care^8,9^. Informing and educating patients regarding AAA and the corresponding risk factors, as well as strategies to mitigate their effects, has long been an important part of postoperative follow-up. However, previous studies have shown that patients who have undergone invasive treatment for AAA often perceived the provided information as insufficient and too infrequently provided^10^.

In other specialty clinical fields, the introduction of specialist nurse-led clinics has proven to be both cost effective and health-promoting, from oncology care^11^ to intensive care^12,13^. While there is literature supporting the beneficial effects of nurse-led clinics on mortality, morbidity, and treatment compliance for other cardiovascular diseases^14^, there is no study that has investigated the potential benefits of a specialist nurse-led clinic within a vascular surgical setting specifically treating patients who have undergone endovascular surgery for AAA. Accordingly, the aim of this study was to investigate the health effects of a postoperative educational intervention performed by specialist nurses at a nurse-led clinic intended to improve the outcomes for patients who underwent endovascular treatment for AAA.

## Methods

### Study design

To answer the study hypothesis that a nurse-led clinic led by specialist nurses could improve the patient’s postoperative recovery, health, and quality of life, a prospective open-label randomized control study design was utilized. The study also followed CONSORT guidelines^15^. The study was conducted at a vascular surgical unit in a Swedish tertiary care university hospital which receives approximately 800 inpatient admissions each year. All patients that met the inclusion criteria were informed about the study prior to surgery during an enrollment phone call. These calls were made in the order in which the patients were scheduled for the procedure by the operation coordinator. Patients that consented to participate in the study were then randomized to one of the two treatment groups via lottery tickets drawn from a non-transparent bowl, and they then received additional participant information both verbally and in writing.

The control group received the standard postoperative follow-up program at the vascular surgical unit, including hospital discharge information provided by a physician and a return visit to a vascular surgeon in the vascular outpatient clinic at one month and one year following surgery. Both the control and intervention groups also underwent routine follow-up CT angiography to identify potential endoleaks or other potential problems with the vascular reconstruction. Study data was obtained at baseline (before the EVAR procedure), as well as at one, six, and 12 months after the procedure.

### Inclusion and exclusion criteria

Adult patients (older than18 years of age) were eligible for the trial if they were able to read and write in Swedish with no cognitive impairment and if they presented with an intact infrarenal aortic aneurysm treatable with a conventional EVAR procedure in an elective setting. In situations where patients had a preliminary plan for an EVAR procedure but then the vascular surgeon ultimately decided to recommend open AAA surgery, these patients were excluded from the study before randomization.

### Description of postoperative standard care

The standard postoperative follow-up program at the vascular surgical unit consisted of hospital discharge information provided by a physician, and a return visit to a vascular surgeon in the vascular outpatient clinic at one month and one year following surgery. Both visits were preceded by routine follow-up CT angiography to identify potential endoleaks or other potential problems with the vascular reconstruction. During both visits to the vascular outpatient clinic, the vascular surgeon enquired about the patient’s postoperative recovery and current sense of health, informed the patient of the radiographic result from the CT-angiography, and gave opportunity for the patient to ask questions regarding their continued recovery and rehabilitation. The standard follow-up program did not include a visit to a specialist nurse. Both the control group and the intervention group received this standard follow-up program.

### Description of the intervention

In addition to the postoperative standard care, the patients in the intervention group received specialist nurse-lead consultation, within two weeks as well as six months after the vascular intervention, during which the participant was offered individual motivational support and education about their disease. The intervention design was based on evidence from previous studies regarding nurse-led clinics in other clinical areas^11–13^, as well as aggregated information regarding EVAR patients’ needs, concerns, and known risk factors^3–5^. The intervention consultation therefore included the following components:

1. The consultation started with an open question to give the participant opportunity to talk about their experiences: “What do you know of the procedure you have received?”
2. Discussion was directed toward lifestyle issues (including physical activity, nutrition, obesity, and tobacco and alcohol use) based on the individual participant needs. The specialist nurse informed the participant about how these lifestyle factors impact the development of atherosclerosis, which in turn could affect the patient’s overall health. The specialist nurse also offered tobacco cessation support if the patient was a smoker.
3. To facilitate an increased understanding of the vascular procedure, the specialist nurse utilized visual aids including an educational movie about vascular anatomy, anatomical models of normal as well as atherosclerotic blood vessels, and the patient’s own x-ray images.
4. At the end of the consultation, the specialist nurse and the patient collaborated to produce written documentation of the conversation, highlighting the individual patient story related to physical activity, nutrition and diet, obesity, and the risks of consuming tobacco and alcohol.

### Data collection

Data was collected in accordance with a pre-defined variable list and compiled in a study database created in Microsoft® Excel® for Microsoft 365MSO (16.0.14326.20900) 64-bit version. Patient demographics collected before surgery included age, body mass index, gender identity, highest educational level, vocational status, smoking habits, current social housing situation, and previous medical history.

### Study endpoints

To measure health outcomes and potential effects of the intervention, two validated self-reported questionnaires were used alongside a set of complementary intervention-specific self-reported items:

1. Health Education Impact Questionnaire (HeiQ) is a generic instrument (40 items), that measures health effects after education^16^. The instrument’s generic nature makes it suitable for use in a wide range of diseases and clinical settings, and it is considered to be user-friendly and psychometrically robust^17^. It is translated and validated to Swedish^18^. The instrument consists of eight scales that measure different perceived health-related aspects: 1. health directed activities, 2. positive and active engagement in life, 3. emotional distress, 4. self-monitoring and insight, 5. Constructive attitudes and approaches, 6. skills and technique acquisition, 7. social integration and support, and 8. health service navigation. The response options for each item are scored 1–4: “strongly disagree,” “disagree,” “agree” and “strongly agree.” The HeiQ-scales are scored by summarizing within scale item scores, followed by dividing the sum with the number of scale items. A higher number represents better outcomes in all scales except for the emotional distress scale, where a higher score represents worse outcome.
2. EuroQol Five Dimension is a standardized instrument designed to measure health outcomes that also has been commonly utilized in health economic analysis^19^. An overall classification of perceived health is measured with a visual analog scale where 0 represents the worst possible imaginable health and 100 represents the best possible imaginable health. Health is further classified in five different dimensions: 1. mobility, 2. self-care, 3. daily activities, 4. pain/discomfort, and 5. depression/anxiety. These dimensions can be graded differently depending on what iteration of the instrument is being used. The present study used the three-scale step level version (EQ-5D-3L), with the alternatives “no problems,” “some/moderate problems,” and “extreme problems” for each dimension.
3. Four complementary items were used to further evaluate the effects of the intervention in the form of visual analog scales: 1. degree of perceived understanding of the diagnosis (1 = not at all and 100 = understands completely), 2. degree of perceived understanding of the purpose of postoperative medical imaging examinations (1 = not at all and 100 = understands completely), 3. degree of desire for additional information regarding their disease (0 = no, it is enough and 100 = yes, much more), 4. degree of perceived concern about treated AAA (0 = never and 100 = all the time).

### Statistical analysis

Median and range were calculated for continuous variables, whereas categorical variables were described in absolute and relative frequencies. For comparison of continuous variables between groups, the Mann–Whitney U test was used. Comparisons of categorical variables between groups were performed with the Fischer’s exact test and Fischer-Freeman-Halton exact test, as appropriate. The Friedman test was used for comparisons within each group from baseline measurements to 12 months following vascular intervention. The plan was to enroll a total of 100 patients for randomization to act as a pilot test of the study, significance was assumed at *p* < 0.05. Statistical analyses were performed with IBM SPSS Statistics software version 28 (IBM Corp. Armonk, NY, USA).

### Ethics approval and consent to participate

This study was approved by the Swedish Regional Ethical Board (registration number: 064-13, was registered in the Swedish national database Researchweb, (01/11/2015 registration-ID VGFOUGSB-579211) as well as retrospectively registered in ClinicalTrials.gov (19/05/2025 NCT06986252). The patients received both written and verbal information before providing written informed consent. All study procedures were carried out in accordance with the Declaration of Helsinki^20^. All patients were informed about the purpose of the study, assured of confidentiality, and provided written consent prior to participation. Participation was voluntary, and patients could withdraw at any time without consequence.

## Results

**Fig. 1:**
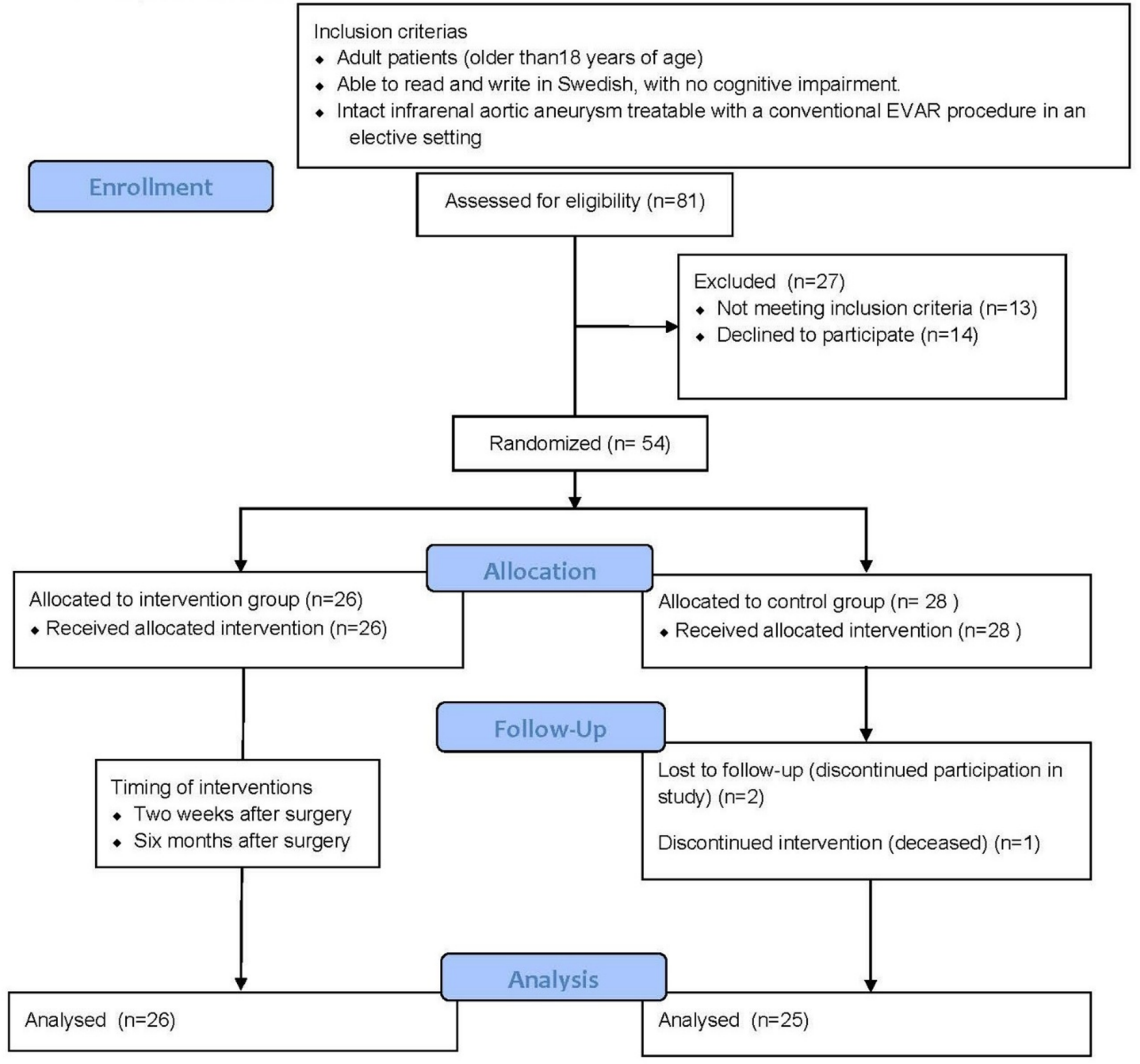
Consolidated standards of outcome reporting in clinical trials (CONSORT) flow chart describing the flow of patients in the trial.

The study was conducted from April 2015 to May 2019. In total, 81 potential participants with AAA were screened, out of which 27 were non-eligible. 54 patients were subsequently randomized, with 26 assigned to the interventional group and 28 to the control group (Fig. 1). Further recruitment of study participants was prevented due to loss of specialist nurses necessary for the intervention.

Mean age in the study population was 75.0 (± SD 6.94), 14.8% were women, and 9.3% had never smoked. 17% of participants had postgraduate education. Table 1 displays the demographic data and comorbidities per group. There were no statistically significant differences between groups in terms of demographic data and clinical characteristics (Table 1).

**Table 1:** Baseline demographic data and clinical characteristics in the study population.

	Intervention group (n = 26)	Control group (n = 25)	*p*-Value^†^
	Median range	Median range	
Age (years)	74 ± 23	76 ± 29	0.445
Body mass index	25.88 ± 23.43	28.16 ± 18.72	0.149
	**n (%)**	**n (%)**	
Male sex	21 (81)	22 (88)	0.703
Never smoker	1 (4)	3 (12)	0.203
Active smoker	8 (32)	4 (12)	0.472
Cardiovascular disease	9 (35)	6 (24)	0.541
Diabetes	1 (4)	4 (16)	0.191
Cancer	2 (8)	2 (8)	1.000
Depression	3 (12)	0	0.235
Postgraduate/University education	4 (15)	4 (16)	0.466
Co-habitant with partner	14 (54)	14 (56)	0.154
Widower/widow	5 (19)	8 (33)	0.154
Retired	23 (89)	19 (76)	0.768

^†^Fischer’s exakt test (*p*-value 0.05) used for categorical variables, Independent Mann–Whitney U-test (*p*-value 0.05) used for continuous variables.

### Health education impact questionnaire - HeiQ

Mean scores in the eight subscales of HeiQ increased from baseline to 12 months after surgery. There was statistical significance at baseline for the subscales positive and active engagement in life (*p* = 0.005, Table 2), self-monitoring and insight (*p* = 0.022, Table 2) and emotional distress (*p* = 0.030, Table 3). Comparisons between groups over time showed only temporary changes in four of the eight subscales, with no clear pattern of favorable results in the intervention group versus the control group, and no significant differences in any subscales were observed at the final measurement point 12 months after EVAR (Tables 2 and 3). Regarding intragroup changes up to one year, no significant changes over time were observed for any of the eight subscales in the control group, whereas the subscale self-monitoring and insight reached nominal significance in the intervention group (*p* = 0.048, Table 2).

**Table 2:** Health Education Impact Questionnaire – Subscale mean score over time between intervention group and control group, as well as internal change over time.

Time of measurement	Intervention group (n = 26)	Control group (n = 25)	*p*-Value^†^
	Median Range	Median Range	
Health directed activities			
Before surgery	2.87 ± 3.00	3.00 ± 2.00	0.381
Postoperative 1 month	2.50 ± 3.00	3.00 ± 3.00	0.024*
Postoperative 6 months	3.00 ± 3.00	3.25 ± 2.00	0.181
Postoperative 12 months	2.62 ± 3.00	3.00 ± .2.50	0.077
*p*-Value‡	0.389	0.824	
Positive and active engagement in life			
Before surgery	2.80 ± 2.20	3.00 ± 1.60	0.005*
Postoperative 1 month	2.80 ± 2.20	3.00 ± 2.00	0.009*
Postoperative 6 months	2.80 ± 3.00	3.00 ± 1.80	0.046*
Postoperative 12 months	2.80 ± 2.80	3.00 ± 2.00	0.135
*p*-Value‡	0.303	0.356	
Self-monitoring and insight			
Before surgery	2.83 ± 2.67	3.16 ± 1.17	0.022*
Postoperative 1 month	3.00 ± 1.50	3.16 ± 1.50	0.247
Postoperative 6 months	3.16 ± 2.67	3.00 ± 1.50	0.655
Postoperative 12 months	3.00 ± 2.17	3.00 ± 1.50	0.739
*p*-value‡	0.048*	0.586	
Constructive attitudes and approaches			
Before surgery	3.00 ± 2.00	3.20 ± 2.00	0.200
Postoperative 1 month	3.20 ± 1.80	3.00 ± 1.60	0.849
Postoperative 6 months	3.20 ± 2.40	3.00 ± 1.80	0.797
Postoperative 12 months	3.10 ± 2.20	3.20 ± 1.40	0.236
*p*-value‡	0.543	0.687	

^†^Independent Mann–Whitney U-test (*p*-value 0.05).

^‡^Friedmans exact test (*p*-value 0.05).

Scoring 1–4, higher scoring represents better outcome.

**Table 3:** Health Education Impact Questionnaire – Subscale mean score over time between intervention group and control group, as well as internal change over time.

Time of measurement	Intervention group (n = 26)	Control group (n = 25)	*p*-Value^†^
	Median Range	Median Range	
Skill and technique acquisition			
Before surgery	3.00 ± 3.00	3.00 ± 1.75	0.936
Postoperative 1 month	2.75 ± 2.25	2.75 ± 2.25	0.560
Postoperative 6 months	2.75 ± 2.25	3.00 ± 1.83	0.137
Postoperative 12 months	2.87 ± 2.50	3.00 ± 2.25	0.521
*p*-Value‡	0.821	0.205	
Social integration and support			
Before surgery	3.20 ± 2.40	3.20 ± 1.60	0.977
Postoperative 1 month	3.20 ± 2.00	3.20 ± 1.50	0.543
Postoperative 6 months	3.20 ± 2.60	3.20 ± 2.20	0.298
Postoperative 12 months	3.00 ± 2.75	3.20 ± 1.60	0.209
*p*-Value‡	0.280	0.724	
Health service navigation			
Before surgery	3.30 ± 1.50	3.20 ± 2.20	0.710
Postoperative 1 month	3.10 ± 2.60	3.20 ± 1.80	0.932
Postoperative 6 months	3.10 ± 2.60	3.20 ± 2.60	0.635
Postoperative 12 months	3.20 ± 2.80	3.00 ± 1.80	0.243
*p*-Value‡	0.100	0.467	
Emotional distress			
Before surgery	1.81 ± 3.00	1.50 ± 1.83	0.030*
Postoperative 1 month	1.83 ± 3.00	1.33 ± 2.00	0.107
Postoperative 6 months	2.16 ± 2.83	1.50 ± 2.00	0.013*
Postoperative 12 months	1.50 ± 3.00	1.50 ± 2.00	0.507
*p*-Value‡	0.355	0.823	

^†^Independent Mann–Whitney U-test (*p*-value 0.05).

^‡^Friedmans exact test (*p*-value 0.05).

Scoring 1–4, higher score represents better outcome in all subscales except Emotional distress, where higher score represents worse outcome.

### Euroqol five dimension three level–EQ-5D-3L

**Fig. 2:**
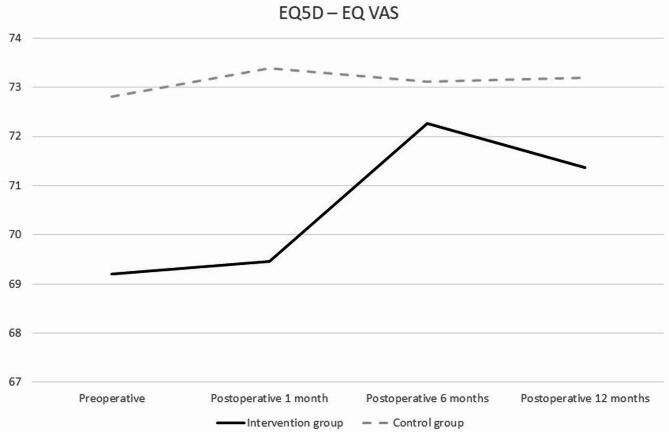
Longitudinal diagram of perceived overall health, for the intervention group and the control group.

Both groups rated their overall health as good throughout the study period (Fig. 2). There was no statistical significance between or within group differences from baseline to 12 months after surgery (Table 4). Also, there were no between or within- group differences noted for any of the five EQ5D-3L domain scores, except in the mobility subscale, where a higher proportion of the intervention group reported some difficulty with mobility from baseline to one year after EVAR (+ 22%, *p* = 0.05, Table 4).

**Table 4:** Proportions (%) reporting levels within EQ5D dimensions: before surgery, six months and 12 months after surgery.

Before and after surgery	Mobility			Self-care			Usual activities			Pain / discomfort			Anxiety / depression		
	Before	6 months	12 months	Before	6 months	12 months	Before	6 months	12 months	Before	6 months	12 months	Before	6 months	12 months
Intervention group															
No problems	68.0	53.8	45.8	100	96.2	95.8	92.0	86.4	91.7	33.3	23.1	20.8	66.7	57.7	70.8
Some/moderate problems	32.0	46.2	54.2	0	3.8	4.2	4.0	11.5	4.2	62.5	73.1	79.2	29.2	38.5	29.2
Extreme problems	0	0	0	0	0	0	4.0	3.8	4.2	4.2	3.8	0	4.2	3.8	0
N =	25	26	24	25	26	24	25	26	24	24	26	24	24	26	24
Reporting some problems	32.0	46.2	54.2	0	3.8	4.2	8.0	15.3	8.4	66.7	76.9	79.2	33.4	42.3	29.2
% change reporting problems 12 months after surgery	+ 22.2^†^			+ 4.2			+ 0.4			+ 12.5			− 4.2		
Control group															
No problems	39.1	52.0	54.2	95.8	96.0	96.0	95.8	84.6	92.0	59.1	44.0	40.0	63.6	76.0	68.0
Some/moderate problems	60.9	48.0	45.8	4.2	4.0	4.2	4.2	12.0	8.0	36.4	56.0	60.0	36.4	24.0	32.0
Extreme problems	0	0	0	0	0	0	0	0	0	4.5	0	0	0	0	0
N =	23	25	24	24	25	24	24	25	25	22	25	25	22	25	25
Reporting some problems	60.9	48.0	45.8	4.2	4.0	4.2	4.2	12.0	8.0	40.9	56.0	60.0	36.4	24.0	32.0
% change reporting problems 12 months after surgery	− 15.1			0			+ 3.8			+ 19.1			− 4.4		

^†^Significant difference over time using Friedmans exact test (*p*-value 0.05).

### Complementary items

No significant differences between groups were observed over time regarding the four complementary items. There were some significant changes within each group over time (Table 5). There was a significant difference at baseline regarding concerns about treated AAA (*p* = 0.028, Table 5). Both groups reported a consistently high understanding of the diagnosis as well as understanding of the radiographic images, but only the control group showed significant improvement over time in terms of understanding the diagnosis. A significant decrease in concerns for treated AAA was shown in both groups over time (Table 5). Both groups desired more information regarding their disease over time, with the intervention group presenting a smaller increase from baseline to 12 months after surgery (+ 6%) compared to the control group (+ 20%), however this was not statistically significant.

**Table 5:** Complementary items.

Time of measurement	Intervention group (n = 26)	Control group (n = 25)	*p*-Value^†^
	Median Range	Median Range	
Understanding the diagnosis			
Before surgery	86.00 ± 85	90.00 ± 72	0.620
Postoperative 1 month	93.00 ± 74	87.00 ± 80	0.212
Postoperative 6 months	90.00 ± 85	93.00 ± 82	0.493
Postoperative 12 months	92.00 ± 70	92.00 ± 40	0.316
*p*-Value‡	0.882	0.022*	
Understanding the radiographic picture			
Before surgery	90.00 ± 64	91.50 ± 72	0.470
Postoperative 1 month	97.00 ± 73	90.00 ± 65	0.062
Postoperative 6 months	92.00 ± 75	93.00 ± 33	0.652
Postoperative 12 months	93.00 ± 26	93.00 ± 35	0.7048
*p*-Value‡	0.752	0.865	
Desire for more information			
Before surgery	21.50 ± 90	21.50 ± 78	0.274
Postoperative 1 month	40.00 ± 96	35.00 ± 100	0.873
Postoperative 6 months	59.50 ± 99	43.00 ± 99	0.822
Postoperative 12 months	37.00 ± 88	39.50 ± 100	0.459
*p*-Value‡	0.096	0.567	
Concerns about treated abdominal aortic anerysm			
Before surgery	59.00 ± 92	39.00 ± 81	0.028*
Postoperative 1 month	26.00 ± 98	24.50 ± 80	0.749
Postoperative 6 months	27.50 ± 92	18.00 ± 84	0.335
Postoperative 12 months	28.00 ± 89	19.00 ± 75	0.197
*p*-Value‡	0.005*	0.008*	

^†^Independent Mann–Whitney U-test (*p*-value 0.05).

^‡^Friedmans exact test (*p*-value 0.05).

Understanding the diagnosis VAS; 0 = Not at all and 100 = Understand completely.

Understanding purpose of X-ray controls VAS; 0 = Not at all and 100 = Understand completely.

Desiring more information VAS; 0 = No, it’s enough and 100 = Yes, much more.

Worry about treated AAA VAS; 0 = Never and 100 = All the time.

## Discussion

The aim of this study was to investigate whether a specialist nurse-led intervention that included a tailored educational program on AAA and its risk factors could improve experience of health, and health-related quality of life in AAA patients undergoing elective EVAR. No statistically significant differences between groups were observed over time. This was a surprising result, as the authors had speculated that the intervention would have a broader effect on several subscales, as was seen in previous studies conducted in other healthcare settings including cardiovascular specialties such as heart failure^21^.

Participants in both groups reported high baseline levels of perceived health and self-management, suggesting a relatively well-functioning population with limited scope for measurable improvement. Consistent with this, both groups demonstrated reduced concern regarding AAA at one year, indicating that standard care alone may sufficiently address patient needs compared to the intervention in its present design.

A notable finding was the higher level of emotional distress in the intervention group at one and six months. While the intervention aimed to improve understanding of disease and modifiable risk factors, the results suggest that increased informational input may have had unintended effects. Specifically, repeated emphasis on risk may have heightened perceived vulnerability without adequately supporting patients’ ability to contextualise this information.

The timing of increased distress further suggests that patients may be particularly sensitive to informational input during the early and intermediate postoperative phases. During these periods, the patients have transitioned from hospital-based structured care to greater self-management responsibility, which may increase a sense of uncertainty. Interventions delivered during this phase might therefore require careful calibration in terms of content, timing, and repetition. Evidence indicating that longer intervals between educational sessions may improve understanding supports this interpretation^22^.

The absence of measurable effects may also reflect the characteristics of AAA and EVAR. In comparison to open repair of AAA, EVAR often require substantially smaller incisions and scarring, resulting in faster postoperative recovery, and it is generally perceived as less traumatic by the patient^23^. Unlike other cardiovascular diseases that produce symptoms, such as activity-induced chest pain or dyspnea, which may interfere with the patient’s daily life, the diagnosis AAA often lacks any concrete physical symptoms^24^.

The intervention group showed a nominal improvement over time in self-monitoring, disease insight, and self-management, while no similar trend was observed in the control group. However, these findings must be interpreted in light of the small sample size and baseline imbalances, both of which limit the reliability of between-group comparisons. As such, the observed changes cannot be considered conclusive. Nonetheless, the consistent direction of improvement within the intervention group may indicate a signal of effect, aligning with previous evidence from nurse-led educational interventions in both general and cardiovascular settings^25,26^. Further adequately powered studies are required to determine whether these trends reflect true intervention effects.

There are methodological aspects that must be considered when interpreting the results of the study. While EQ-5D-3L is a validated and widely used instrument for investigating health outcomes, it is not designed to detect subtle psychological changes that might occur in this study population. HeiQ has a similar methodological challenge, though it is designed to investigate health effect after education, and has proven to be both psychometrically robust and user-friendly, it may not fully capture transient distress related to information processing. Combined with the small sample size, this increases the likelihood that small but clinically relevant effects were not detected. Finally, the difference in several baseline values must be taken into consideration. The authors hypothesize that a larger sample could have resulted in more equal baseline values between the two groups but must also acknowledge the possibility that the RCT-study in itself might have unintentionally affected some of the self-reported baseline values by introducing expectancy or framing effects. Patients who were informed that they would receive an intervention may have rated their condition more positively due to anticipated support, whereas those assigned to the control group may have reported lower perceived capability or understanding. Conversely, awareness of being in an intervention group may also have increased self-scrutiny, potentially lowering self-ratings. These potential response shifts highlight a limitation of self-reported measures in unblinded RCTs and should be considered when interpreting the baseline differences.

### Strengths and limitations

This study has several methodological strengths. The randomized controlled design enhances internal validity and reduces the likelihood of systematic bias between groups. The low attrition rate supports the completeness and consistency of follow-up data, strengthening confidence in the observed patterns over time. Furthermore, the included participants clinical and demographic characteristics were to a high degree representative of the Swedish patient population^27^. Of the 27 patients not eligible for participation in the study, 13 were due to change in recommended surgical intervention from initial endovascular to open surgery, and 14 due to declining participation. While we did not investigate the clinical reasoning behind the altered recommendation, nor motives for declining participation in the study, a contributing factor might be complex comorbidity and perioperative concerns.

However, important limitations must be acknowledged. The early termination of the trial resulted in a substantially reduced sample size, limiting statistical power and increasing the probability of type II error. Consequently, the absence of statistically significant differences between groups should be interpreted with caution, as small but meaningful effects may not have been detected. The high baseline levels of perceived health and self-management further suggest a ceiling effect, reducing the sensitivity of the study to detect improvement. The single-centre design limits external validity, as findings may be influenced by local organisational structures and the quality of standard care. In this study, standard care appeared to be comprehensive, which may have reduced the potential added value of the intervention. In addition, the intervention was not developed in collaboration with patients, which may have contributed to suboptimal alignment with patient needs and preferences. This may partly explain why the intervention did not produce the intended benefits and may have contributed to increased psychological burden. A further limitation is the absence of detailed process evaluation. Without data on how participants engaged with the intervention, it is difficult to determine whether the observed outcomes reflect limitations in the intervention itself or in its implementation.

### Future research

Future research should incorporate process evaluations, explore optimal timing and intensity of information delivery, and consider the use of more sensitive, condition-specific outcome measures. The use of disease-specific questionnaires, alongside validated instruments targeting psychological constructs such as anxiety, emotional distress, and cognitive responses to risk information, may improve the ability to detect subtle but clinically relevant changes that are not captured by generic measures.

## Conclusions

This study did not demonstrate a measurable benefit of a specialist nurse-led educational intervention over standard care following EVAR for AAA. While small improvements in self-monitoring and disease insight were observed, these effects were limited and did not translate into broader or sustained improvements in health-related outcomes. In contrast, the improvement in perceived understanding within the control group suggests that standard care, combined with time for reflection, may be sufficient for many patients.

The finding of increased emotional distress in the intervention group raises important concerns regarding the potential unintended effects of intensified educational approaches. These results challenge the assumption that increased informational input is inherently beneficial and underscore the need to balance informational support with psychological impact. The timing and delivery of education appear to be critical factors influencing patient outcomes. Future studies should move beyond simply increasing informational content and instead focus on how, when, and for whom education is delivered. This includes exploring adaptive, patient-centred approaches, as well as evaluating interventions in different AAA populations, such as those undergoing open repair or conservative management. Greater attention should also be given to outcome measures capable of capturing short-term psychological responses to healthcare interventions.

### Relevance for clinical practice

The findings suggest that adding structured educational interventions to standard care may provide limited additional benefit in this patient group. Specialist nurse-led support may still be valuable for promoting self-management, but interventions should be carefully tailored with targeted, stage-appropriate information, and attention to timing, patient readiness and potential psychological effects.

## Data Availability

The datasets used during the current study are available from the corresponding author on reasonable request.

## Abbreviations

AAA: Abdominal aortic aneurysm
EVAR: Endovascular aortic repair
EQ-5D-3L: Euroqol five dimension three level
HeiQ: Health Education Impact Questionnaire
